# The impact of serotype-specific vaccination on phylodynamic parameters of *Streptococcus pneumoniae* and the pneumococcal pan-genome

**DOI:** 10.1371/journal.ppat.1006966

**Published:** 2018-04-04

**Authors:** Taj Azarian, Lindsay R. Grant, Brian J. Arnold, Laura L. Hammitt, Raymond Reid, Mathuram Santosham, Robert Weatherholtz, Novalene Goklish, Claudette M. Thompson, Stephen D. Bentley, Katherine L. O’Brien, William P. Hanage, Marc Lipsitch

**Affiliations:** 1 Center for Communicable Disease Dynamics, Department of Epidemiology, T.H. Chan School of Public Health, Harvard University; Cambridge, Massachusetts, United States of America; 2 Center for American Indian Health, Johns Hopkins Bloomberg School of Public Health, Baltimore, Maryland; United States of America; 3 Wellcome Trust Sanger Institute, Cambridge, United Kingdom; University of Oxford, UNITED KINGDOM

## Abstract

In the United States, the introduction of the heptavalent pneumococcal conjugate vaccine (PCV) largely eliminated vaccine serotypes (VT); non-vaccine serotypes (NVT) subsequently increased in carriage and disease. Vaccination also disrupts the composition of the pneumococcal pangenome, which includes mobile genetic elements and polymorphic non-capsular antigens important for virulence, transmission, and pneumococcal ecology. Antigenic proteins are of interest for future vaccines; yet, little is known about how the they are affected by PCV use. To investigate the evolutionary impact of vaccination, we assessed recombination, evolution, and pathogen demographic history of 937 pneumococci collected from 1998–2012 among Navajo and White Mountain Apache Native American communities. We analyzed changes in the pneumococcal pangenome, focusing on metabolic loci and 19 polymorphic protein antigens. We found the impact of PCV on the pneumococcal population could be observed in reduced diversity, a smaller pangenome, and changing frequencies of accessory clusters of orthologous groups (COGs). Post-PCV7, diversity rebounded through clonal expansion of NVT lineages and inferred in-migration of two previously unobserved lineages. Accessory COGs frequencies trended toward pre-PCV7 values with increasing time since vaccine introduction. Contemporary frequencies of protein antigen variants are better predicted by pre-PCV7 values (1998–2000) than the preceding period (2006–2008), suggesting balancing selection may have acted in maintaining variant frequencies in this population. Overall, we present the largest genomic analysis of pneumococcal carriage in the United States to date, which includes a snapshot of a true vaccine-naïve community prior to the introduction of PCV7. These data improve our understanding of pneumococcal evolution and emphasize the need to consider pangenome composition when inferring the impact of vaccination and developing future protein-based pneumococcal vaccines.

## Author summary

Pneumococcal disease caused by the bacteria *Streptococcus pneumoniae* remains a significant cause of morbidity and mortality despite the existence of an effective vaccine. This is because the vaccines only target a small proportion of the total pneumococcal population. Introduction of vaccine in the United States removed vaccine serotypes leaving an open niche that was rapidly filled by non-vaccine serotypes. Forecasting which serotypes, and more generally which pneumococcal lineages, will increase in frequency in carriage and disease is an active area of research with significant public health importance. Here, we investigate the evolutionary impact of vaccination on the pneumococcal population using genomic data from a collection of 937 pneumococcal isolates collected from 1998–2012 among Native American communities. We find the impact of vaccine on the pneumococcal population could be observed in reduced diversity and changing frequencies of genes. Diversity subsequently rebounded through expansion and in-migration of non-vaccine lineages. Further, frequencies of genes coding for protein antigens important to host-pathogen interaction were initially disrupted but later returned to pre-vaccine values, suggesting selection may have acted in maintaining frequencies. These data improve our understanding of pneumococcal evolution and emphasize the need to consider genome composition when inferring the impact of vaccination.

## Introduction

Pneumococcal conjugate vaccines (PCV) target capsular serotype-specific polysaccharides of the respiratory pathogen *Streptococcus pneumoniae*, which causes substantial morbidity and mortality [1,2]. Since the heptavalent PCV and 13-valent PCV were introduced in the United States (US) in 2000 and 2010, respectively, their effectiveness in reducing pneumococcal carriage and invasive disease has been well documented [3–6]. In communities where PCV has been introduced, the prevalence of vaccine serotypes (VT) in carriage and invasive disease consistently decreases, resulting in an overall reduction in pneumococcal disease. However, in a process called “serotype replacement,” non-vaccine serotypes (NVT) subsequently increase in carriage after vaccine introduction, leading to slight increases in NVT-associated disease in almost all populations where the vaccine is introduced [7,8]. Because polysaccharide serotypes change rarely during pneumococcal evolution, common pneumococcal lineages typically contain only one or a few serotypes. As a result, PCV implementation removes lineages containing only VT from the population, while lineages including both VT and NVT experience genetic bottlenecks [9–11].

Forecasting which serotypes, and more generally which pneumococcal lineages, will increase in frequency in carriage and disease is an active area of research with significant public health importance. For *S*. *pneumoniae*, the most commonly used vaccines globally target a fraction of the more than 93 recognized capsular serotypes [12]. The bacteria’s capsule (CPS) is the most important determinant of virulence and the strongest predictor of prevalence [13], as well as the target of PCVs; thus, changes in CPS serotype frequency have been the focus of many analyses of vaccine effect. However, selection acts on genes outside the operon determining CPS serotype. Whole-genome sequencing data has enabled investigation of variation in multiple genomic loci and genome content among pneumococci, focused on loci involved in host immunity and niche adaption. We focus here on two categories of proteins. The first is antigens (hereafter, when we use the generic term antigen we refer to proteins that elicit an immune response, not to the polysaccharide capsule). Antigens such as pneumococcal surface proteins A and C (*pspA* and *pspC*) and pilus are of specific interest as possible targets for non-capsular polysaccharide based vaccines [14]. Together with other components of the pneumococcal genome, the capsule and non-capsular antigens comprise the overall antigenic profile of a pneumococcus [15–17]. Moreover, evolution among metabolic genes gives rise to distinct metabolic-profiles among pneumococcal lineages, which may be adapted for specific metabolic niches [18,19]. Thus, multiple loci may interface with the host, affecting the overall evolutionary success of a lineage at a population level.

Gene content varies tremendously among pneumococcal lineages [20,21]. The pneumococcal pangenome consists of “core genes” shared by ≥99% of strains and “accessory genes” present at frequencies ≤99%. Accessory genes may include polymorphic antigens, phage and plasmid-related chromosomal islands, and integrative and conjugative elements (ICE) harboring antimicrobial resistance genes. The latter are mobile genetic elements (MGE), which are often acquired through horizontal gene transfer (HGT) and may remain stable in pneumococcal lineages [21]. Variations in gene content among lineages of a bacterial species are associated with ecological niche specialization and are important for adaptation to changing environments, including selection by vaccine-induced and natural host immunity [21–23]. For the pneumococcus, MGE affect the bacteria’s ability to recombine (i.e., competence) [24], antimicrobial susceptibility [25], and carriage duration [26]. Accessory loci may also be acted upon by negative frequency dependent selection (NFDS), hinting at their underlying role in non-serotype-specific immunity and *S*. *pneumoniae* ecology [27]. Taken together, gene variation beyond the capsular polysaccharide loci may significantly impact virulence, fitness, transmission, and, in turn, the overall epidemiology and ecology of pneumococcal strains.

Before PCV introduction, Navajo and White Mountain Apache (N/WMA) Native American communities in the Southwestern US experienced rates of invasive pneumococcal disease (IPD) 2–5 times higher than the general US population [28,29]. Pneumococcal carriage prevalence among N/WMA pre-PCV7 was 50% among all ages and 75% among children <2 years of age, significantly higher than the general population [30]. Thirty-eight percent of all pneumococcal carriage isolates were PCV7 serotypes [30]. After introduction of PCV7, carriage prevalence of PCV7 VT declined, and the rate of IPD among N/WMA caused by VT decreased by 89% [31]. However, carriage prevalence of NVT strains increased, resulting in no overall change in pneumococcal carriage prevalence among children or adults [5]. Also, despite increased NVT carriage there was no corresponding increase in the rate of IPD caused by NVT. After introduction of PCV13 in 2010, carriage of PCV13-specific serotypes declined by 60% among children <5 years of age within the first two years [6]. Yet, overall IPD rates among N/WMA still remain higher than those in the general US population [32].

Here, we analyze a sample of 937 pneumococci collected over 14 years and spanning before, during, and after the introduction of PCV7 and PCV13 vaccines among N/WMA. To understand the evolutionary impact of vaccination and characterize the shift from VT to NVT, we assessed the recombination, evolution, and pneumococcal population history, classified by serotype and by whole-genome sequencing data, across vaccine introduction periods. Furthermore, we investigated metabolic loci variation and pangenome composition over time, with a focus on pneumococcal antigens.

## Methods

### Study population and pneumococcal isolation

This study included pneumococci isolated from a subset of participants of three prospective, observational cohort studies of pneumococcal carriage among N/WMA families described elsewhere (hereafter, “parent” studies) [1,6,30]. Briefly, participants living on reservations in the southwest USA were enrolled during three periods: 1998–2001, 2006–2008 and 2010–2012. Nasopharyngeal (NP) swab specimens were obtained during visits to Indian Health Services (IHS) facilities or the participants’ home to determine pneumococcal carriage status (S1 Fig) [30]. A random subsample of isolates was selected from each time period, with an oversampling of isolates post-PCV7 (S2 Fig). A single isolate was chosen from each participant; however, previous pneumococcal carriage history was not assessed. With the exception of a subset of isolates collected from 2006–2008, all isolates were obtained from children ≤5 years of age.

### DNA sequencing, *de novo* assembly, pangenome, and population structure

Genomic DNA from *S*. *pneumoniae* isolates were sequenced on the Illumina HiSeq, yielding ≥30-fold coverage per isolate. Paired-end 100 bp reads were filtered by quality and length. Serotypes were determined by mapping reads to concatenated CPS locus sequences of 93 pneumococcal serotypes using SRST2 [12,33]. Serotypes for isolates identified as serogroup 6 were further resolved using PneumoCaT [34]. Multilocus sequence type (MLST) was determined through a similar approach using SRST2. *De novo* genome assemblies were generated with Velvet [35] and annotated using Prokka v1.11 [36]. After annotation, the pangenome was analyzed with Roary, and a concatenated alignment of clusters of orthologous genes (COGs) shared among ≥99% of all strains (i.e., core genome) was abstracted [37]. Pneumococcal population structure was assessed using core genome SNPs with hierBAPS, which was run three times using maximum clustering sizes of 20, 40, and 60 [38]. A maximum likelihood (ML) phylogeny was estimated using RAxML v8.1.5 with GTR+Γ nucleotide substitution model and 100 bootstrap replicates [39]. Sequence clusters (SCs) (i.e., lineages) identified using hierBAPS were annotated on the core genome phylogeny. For the study period during which pediatric and adult isolates were collected (2006–2008), the proportion of isolates by SC was compared between age groups to 10,000 random deviates of a Dirichlet distribution [40].

### Reference-based genome assembly and recombination analysis

A subsample of isolates from each SC and 25 publicly available reference genomes were aligned using Parsnp and visualized using Gingr to identify the most appropriate genome for reference-based mapping [41]. The phylogenetically closest genome was selected for reference-based mapping of isolates belonging to that SC. For four out of 27 SCs, a monophyletic match was not available; therefore, we generated references by refining, ordering, and concatenating the best draft assembly in the SC. A second *de novo* assembly was generated with SPAdes and assemblies were then merged using Zorro [42]. After this, SSPACE and GAPFILLER were used to scaffold the assembly and remove Ns [43,44]. Final contigs were ordered using Mauve, manually curated using ACT, and concatenated [45]. Filtered Illumina reads from isolates comprising each SC were mapped to the selected reference using SMALT v0.7.6 and SNPs were identified using SAMtools v1.3.1 [46]. SNPs were filtered requiring a depth of coverage of five and a minimum alternate allele frequency of 0.75. The output was analyzed as previously described to generate whole-genome multiple sequence alignments for each SC [9,47].

Next, we identified recombination among SCs using Gubbins [48]. Gubbins identifies SNPs introduced through recombination and allows censoring for downstream phylogenetic analysis. Results of Gubbins analyses were visualized using Phandango [49]. For SCs in which over 50% of the genome was censored due ancestral recombination events, we either sub-clustered SCs clearly delineated monophyletic clades (e.g., SC19 which was comprised of serotypes 15A and 17F) or removed divergent isolates that were significantly affected by recombination. Sub-clustered SCs were annotated on the ML phylogeny and then reanalyzed with Gubbins.

### Analysis of VT and NVT lineage population dynamics

For comparison between vaccine periods, isolates were subdivided into three epochs and six sub-epochs by year of collection: pre-PCV7 sub-epochs 1A (1998) and 1B (1999–2001); post-PCV7 sub-epochs 2A (2006) and 2B (2007–2008); PCV13 sub-epochs 3A (2010) and 3B (2011–2012) (S2 Fig). Collection years were grouped to balance sample sizes among sub-epochs. To determine the representativeness of the genomic sample to the parent studies from which the sample was drawn, we compared the serotype distribution and serotype diversity (Simpson’s D) of unique carriage isolates from the three parent studies of pneumococcal carriage [1,6,30], by epoch, to that of the sample. Core genome alignments were generated for isolates in each sub-epoch using Roary, and population genomic statistics including Tajima’s *D* [50], Watterson’s estimator (Θ_w_) [51], and nucleotide diversity were calculated for each period using 0-fold and 4-fold degenerate sites. The ratio of diversity at non-synonymous sites to synonymous sites (πN/πS) was also calculated as a measure of selection. The same statistics were calculated for each SC. Code for calculating population genetic statistics using Roary output is available at http://github.com/c2-d2/Projects/NWMA_Pneumo/.

ML phylogenies of SCs, inferred from recombination-censored alignments, were used to test temporal signal by assessing correlation between strain isolation date and root-to-tip distance. SCs with poor root-to-tip correlation were assessed for residual recombination and phylogenetic signal. SCs determined to have sufficient temporal signal were analyzed with BEAST v1.8.2 [52]. For each SC or sub-SC a combination of strict and relaxed molecular clock models and constant and Gaussian Markov random field (SkyGrid) demographic models [53] were tested using recombination-free SNP alignments, ascertainment bias correction [54,55], and HKY nucleotide substitution model. For SCs in which the coefficient of variation for relaxed molecular clock models was high (i.e., significant rate heterogeneity across the tree), a random local clock (RLC) model was also tested [56]. Markov chain Monte Carlo lengths for each model run ranged from 150 million to 1 billion depending on the size of the SC and length of the SNP alignment. MCMC chains were sampled to obtain 10,000 trees and 10,000 parameter estimates in the posterior distribution. Effective sampling size (ESS) values were assessed to determine sufficient mixing using Tracer v1.6.0, and runs with ESS values of 200 for all parameters were accepted. Marginal likelihood estimates (MLE) were obtained for each model using path-sampling and stepping-stone analysis, and models were compared using Bayes Factors [57,58]. Parameter estimates for the evolutionary rate, root height (i.e., TMRCA), and *N*_*e*_ were obtained from the best-fit model. For SCs in which SkyGrid demographic models were fit, the slope of the *N*_*e*_ change over time was calculated to determine directionality, and the 95% highest posterior density (HPD) was used to determine significance.

### Variation in metabolic loci, accessory genome content, and non-capsular antigens among epochs

To assess the impact of PCV7 on the pneumococcal pangenome we compared frequencies of polymorphisms in core genes and accessory genome COGs among sub-epochs, focusing on antigens and metabolic loci for the core genome and on antigens for the accessory genome analysis. We identified metabolic genes using coding sequences found in *S*. *pneumoniae* reference strain D39 (RefSeq: NC_008533.1) that were annotated as “Metabolism” according to KEGG Orthology (KO) groupings of the KEGG database (http://www.genome.jp/kegg/) and were assigned to a known metabolic pathway (KEGG pathway spd01100). Pangenome analysis using Roary was repeated including D39, and COGs found in the core genome (i.e., present among all 937 taxa) with ≥90 BLAST identity to metabolic genes were abstracted. A concatenated alignment of core metabolic COGs was then constructed, and biallelic SNP sites were identified. To assess changes to the accessory genome, we obtained the binary presence-absence matrix of accessory COGs present in frequencies ranging from 5–95% among all taxa. This frequency range was conservatively selected to mitigate the effect of genome assembly and annotation errors in COG identification. Last, we used a previously described method to identify the variants of 19 polymorphic antigens [15]. These antigens have measurable interactions with the host immune system, and therefore are thought to be under the greatest population level host immune pressure. Ten additional antigens were evaluated (*lysM*, *lytB/C*, *pcpA*, *pcsB*, *phtE*, *piaA*, *piuA*, *psaA*, *SP2027*, *pce*) but were excluded because they were deemed nearly monomorphic due to their low nucleotide diversity.

Using the concatenated nucleotide alignment of metabolic loci and a binary presence absence alignment accessory COGs and antigen variants, ML phylogenies were inferred using RAxML with GTRGAMMA (nucleotide) or GTRCAT (binary) substitution model and 100 bootstrap replicates. The cophenetic (patristic) distances of each phylogeny were read into R, and the meandist function in the package *vegan* was used to calculate within-group distances for three population groupings: serogroup, serotype, and SC. Within-group distances for population stratifications were then compared. For each set of genomic loci (metabolic, accessory COGs, and antigens), frequencies were computed for each of the six sub-epochs. Mean squared errors (MSEs) were then calculated to assess changes in frequencies from Epoch 1A. This was done by subsampling 75 individuals with replacement from each sub-epoch and performing 1000 bootstrap replicates of each comparison (e.g., Epoch 1A vs. 1B, 1A vs. 2A, 1A vs. 2B, and so on). The significance of changes in antigen distributions among epochs was additionally tested by comparing the proportion of antigen variants between Epochs 1–3 to 10,000 random deviates of a Dirichlet distribution.

### Ethics statement

The Navajo Nation, White Mountain Apache tribe and the IRBs of the Johns Hopkins Bloomberg School of Public Health, the Navajo Nation and the Phoenix Area IHS approved this study. During the original pneumococcal carriage studies from which these isolates were obtained, written informed consent was obtained from adult participants and from caregivers of child participants. Assent was obtained from children 7–17 years. Isolates were obtained from NP swabs, as previously described, and de-identified for analysis.

## Results

### Population structure

We analyzed genomic data from a total of 937 pneumococcal carriage isolates collected from N/WMA Native Americans in Southwestern US between 1998 and 2012. All isolates were obtained from children ≤5 years of age with the exception of 125 isolates (13.3% of total) collected from individuals 6–76 years of age during 2006–2008. Isolates collected from 1998–2001 (n = 274) were obtained from communities that served as the control for cluster-randomized PCV7 trials and therefore represent a vaccine naïve population. Isolates collected during 2006–2008 (n = 398) represent the post-PCV7 pneumococcal population, and isolates from 2010–2012 (n = 265) were sampled during the implementation of PCV13 (S2 Fig). Whole-genome sequencing data has been deposited in NCBI sequence read archive (SRA) under accession number ERP009399, BioProject PRJEB8327. Individual accession numbers are provided in supplementary file 1.

**Fig 1:**
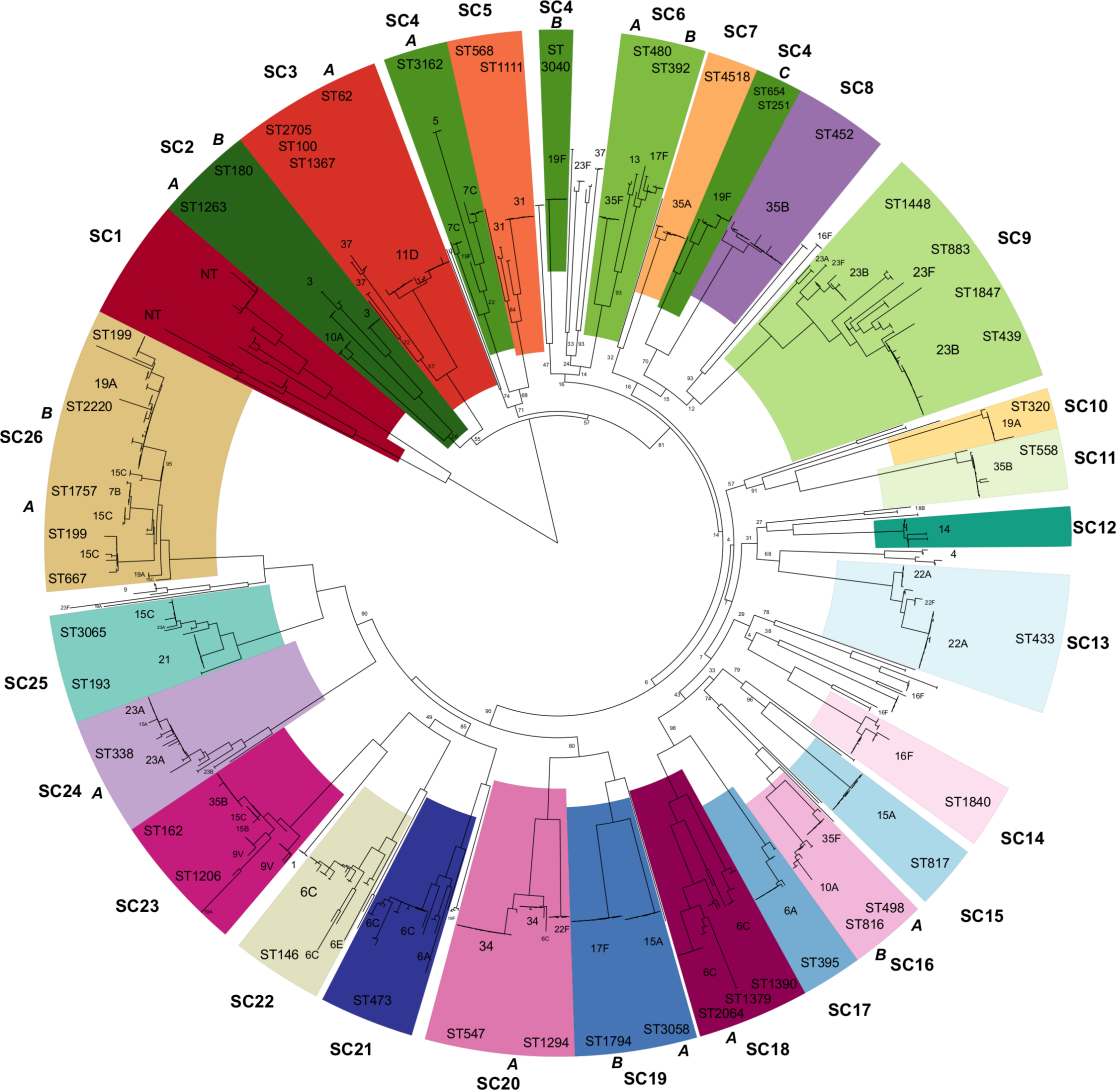
Phylogeny of pneumococcal population structure. Maximum likelihood phylogeny of 937 pneumococcal carriage isolates inferred from an alignment of 1,111 core COGs including 78,525 polymorphic sites using RAxML with GTR+Γ nucleotide substitution model and 100 bootstrap replicates. Clades are colored by sequence cluster (SC), which are labeled on the outside ring. Some SCs are further divided into monophyletic sub-clusters (A, B, C) based on ancestral recombination history. Pneumococcal serotypes and MLST are labeled on each clade and bootstrap values are labeled on internal branches.

Pangenome analysis of *de novo* genome assemblies identified 8,674 COGs, of which 1,111 were present in ≥ 99% of strains (i.e., the core genome). Analysis of population structure using hierBAPS identified 27 SCs, two of which (SC27 and SC4) were polyphyletic in the ML phylogeny (Fig 1). SC27 was comprised of low frequency genotypes whereas SC4 contained three distinct monophyletic clades that were bifurcated by branches with low bootstrap support. Based on recombination analysis using Gubbins and assessment of temporal signal (i.e., molecular clock), SC4 as well as 10 other SCs were further subdivided, as it was evident that substantial ancestral recombination events occurred on branches separating dominant monophyletic clades. This subdivision is consistent with the biological definitions of lineages or sub-populations [59,60]. Subsequent analysis focused on 33 SCs or sub-SCs that varied in size from 10 to 71 isolates (Table 1). The proportion of isolates belonging to each SC differed between age groups for only four of 27 SCs, among isolates collected from 2006–2008. SC07 (serotype 35A) and SC15 (serotype 15A) were more common among children ≤5 years of age, 0.8% and 1.6% adults compared to 3.3% and 4.4% children, respectively (*p* = 0.03 and 0.05). SC08 (serotype 35B) and SC26 (serotypes 19A/15C) were more common among adults, 5.6% and 14.4% adults compared to 2.2% and 8.4% children, respectively (*p* = 0.05 and 0.04).

**Table 1:** Demography and vaccine type composition of pneumococcal sequence clusters. For each sequence cluster (SC) or sub-cluster, the number of isolates, proportion of PCV7 and PCV13 vaccine types, and recombination rate (*r/m*) are listed. The best fit demographic and molecular clock model as determined through comparison of marginal likelihood estimates using BEAST are specified. The *Ne* directionality (constant, exponentially increasing or decreasing) were determined by assessing the slope of the *Ne* during the study period (1998–2012) and 95% Highest Posterior Density (HPD).

SC	Isolates	% PCV7	% PCV13	SNP Sites	Recombination Rate (r/m)	Demographic Model	Clock Model	*Ne* slope (1998–2012)	*Ne* Direction
02-A	19	0.0%	100.0%	4,137	1.49	Constant	Relaxed	-	→
02-B	10	0.0%	0.0%	375	1.17	SkyGrid	Relaxed	0.032	→
03-A	35	0.0%	0.0%	1,048	2.27	SkyGrid	Relaxed	-0.041	→
04-A	19	0.0%	0.0%	1,239	1.65	Constant	Relaxed	-	→
04-B	10	100.0%	0.0%	147	0.01	SkyGrid	Relaxed	-0.027	→
04-C	14	100.0%	0.0%	408	10.31	Constant	Relaxed	-	→
05	21	0.0%	0.0%	1,248	0.93	Constant	Relaxed	-	→
06-A	10	0.0%	0.0%	178	0.04	SkyGrid	Strict	-0.031	→
06-B	11	0.0%	0.0%	679	2.28	SkyGrid	Relaxed	-0.002	→
07	15	0.0%	0.0%	556	1.33	Constant	Relaxed	-	→
08	28	0.0%	0.0%	660	6.09	SkyGrid	Relaxed	-0.020	→
09-A	71	26.8%	0.0%	2,049	4.50	Constant	Relaxed	-	→
10	12	0.0%	100.0%	150	15.03	Constant	Relaxed	-	→
11	18	0.0%	0.0%	370	1.97	SkyGrid	Strict	-0.067	↘
12	13	100.0%	0.0%	293	1.23	Constant	Relaxed	-	→
13	41	0.0%	0.0%	979	2.12	SkyGrid	Relaxed	-0.021	→
14	19	0.0%	0.0%	530	7.99	SkyGrid	Relaxed	0.008	→
15	19	0.0%	0.0%	544	0.05	Constant	Relaxed	-	→
16-A	11	0.0%	0.0%	270	0.36	Constant	Relaxed	-	→
16-B	12	0.0%	0.0%	2,954	2.30	SkyGrid	Relaxed	0.084	→
17	13	0.0%	100.0%	176	0.61	SkyGrid	Random Local	-0.290	↘
18-A	21	0.0%	0.0%	733	5.04	Constant	Relaxed	-	→
19-A	15	0.0%	0.0%	165	0.00	Constant	Relaxed	-	→
19-B	21	0.0%	0.0%	283	0.10	Constant	Relaxed	-	→
20-A	35	0.0%	0.0%	827	3.39	Constant	Random Local	-	→
21	28	0.0%	21.4%	1,561	10.27	Constant	Relaxed	-	→
22	27	0.0%	0.0%	987	14.11	Constant	Relaxed	-	→
23	41	0.0%	2.4%	677	8.24	Constant	Relaxed	-	→
24-A	32	0.0%	0.0%	753	15.00	SkyGrid	Relaxed	0.013	→
25	32	0.0%	0.0%	889	6.33	Constant	Relaxed	-	→
26-AB	84	0.0%	56.0%	2,344	5.40	SkyGrid	Relaxed	-0.059	↘
26-A	40	0.0%	0.0%	923	4.81	SkyGrid	Strict	-0.110	↘
26-B	44	0.0%	100.0%	599	3.83	SkyGrid	Relaxed	-0.020	→
27*	95	18.9%	13.7%	-	-	-	-	-	-

*SC27 is polyphyletic, comprised of several at low frequencies

### Representativeness of sequenced isolates

**Fig 2:**
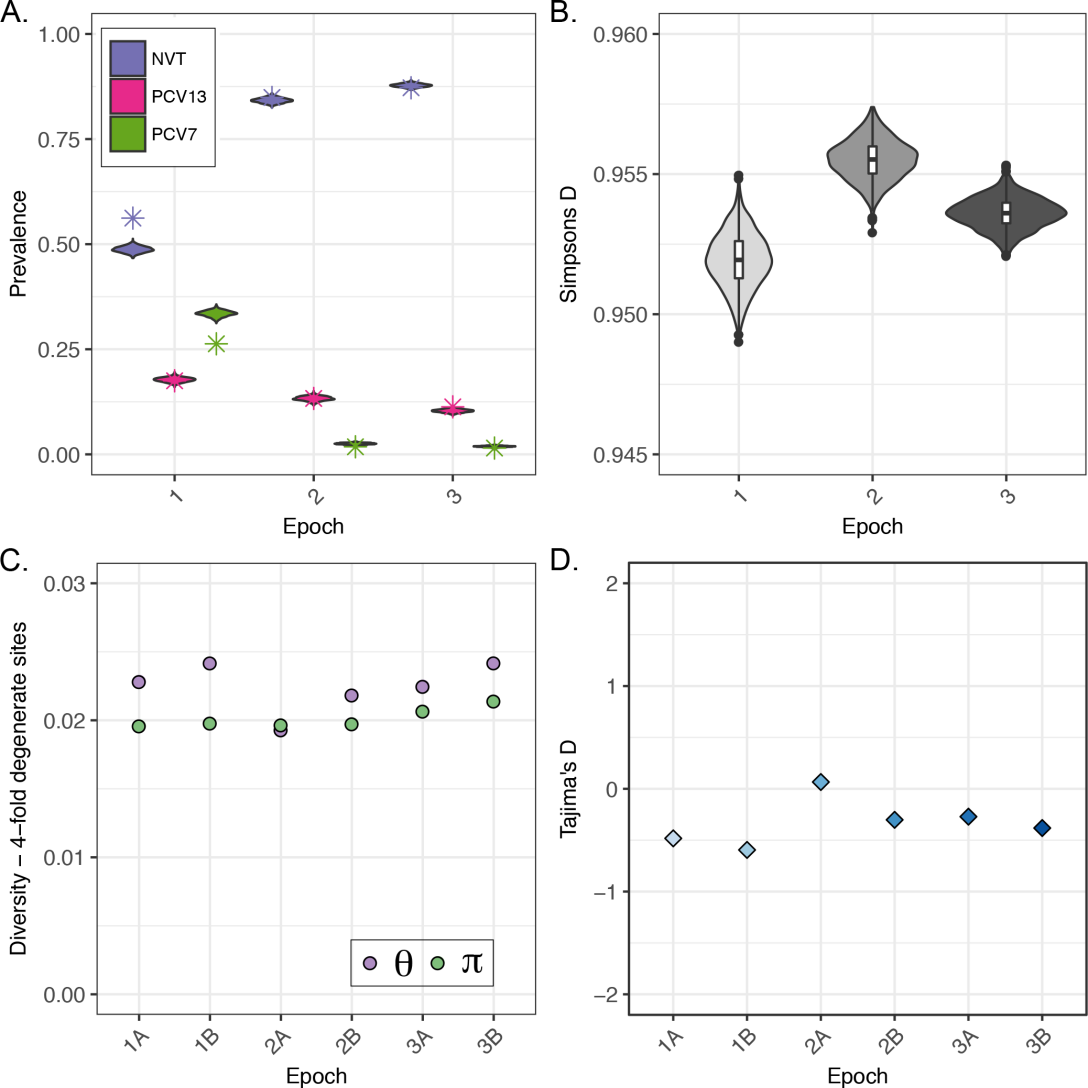
Pneumococcal population dynamics pre- and post-vaccine, 1998–2012. The study periods were subdivided into three epochs and six sub-epochs: Pre-PCV7 [Epoch 1: A (n = 105), B (n = 169)], Post-PCV7 [Epoch 2: A (n = 79), B (n = 319)], and PCV13-Intermediate [Epoch 3: A (n = 119), B (n = 146)]. A.) The proportions of vaccine types (VT) for each epoch from three parent studies (violin) and current study subsample (asterisk). Parent study VT proportions are estimated from serotypes of 3,868 carriage events from previous N/WMA studies [1,6,30]. PCV7 VT include serotypes 4, 6B, 9V, 14, 18C, 19F and 23F. PCV13 vaccine types (minus PCV7 types) include only serotypes 1, 3, 5, 6A, 7F, 19A. Violin plots represent the realization of 1000 bootstrap replicates subsampling with replacement from each epoch. Asterisks represent the point estimates for VT proportions in the current study. B.) Simpson’s Diversity Index of serotype diversity across study periods estimated from three parent studies (n = 3,868). This measure summarizes the number and abundance of each serotype. C.) Population genetic measures of diversity, Watterson estimator (Θ_w_) and π (average number of pairwise differences), estimated from 4-fold (synonymous) degenerate sites of taxa in sub-epochs of the current study. D.) Population genetic statistic Tajima’s D estimated from the core genomes of taxa in sub-epochs of the current study. Negative values of Tajima’s D indicate many sites with a rare minor alleles.

**Fig 3:**
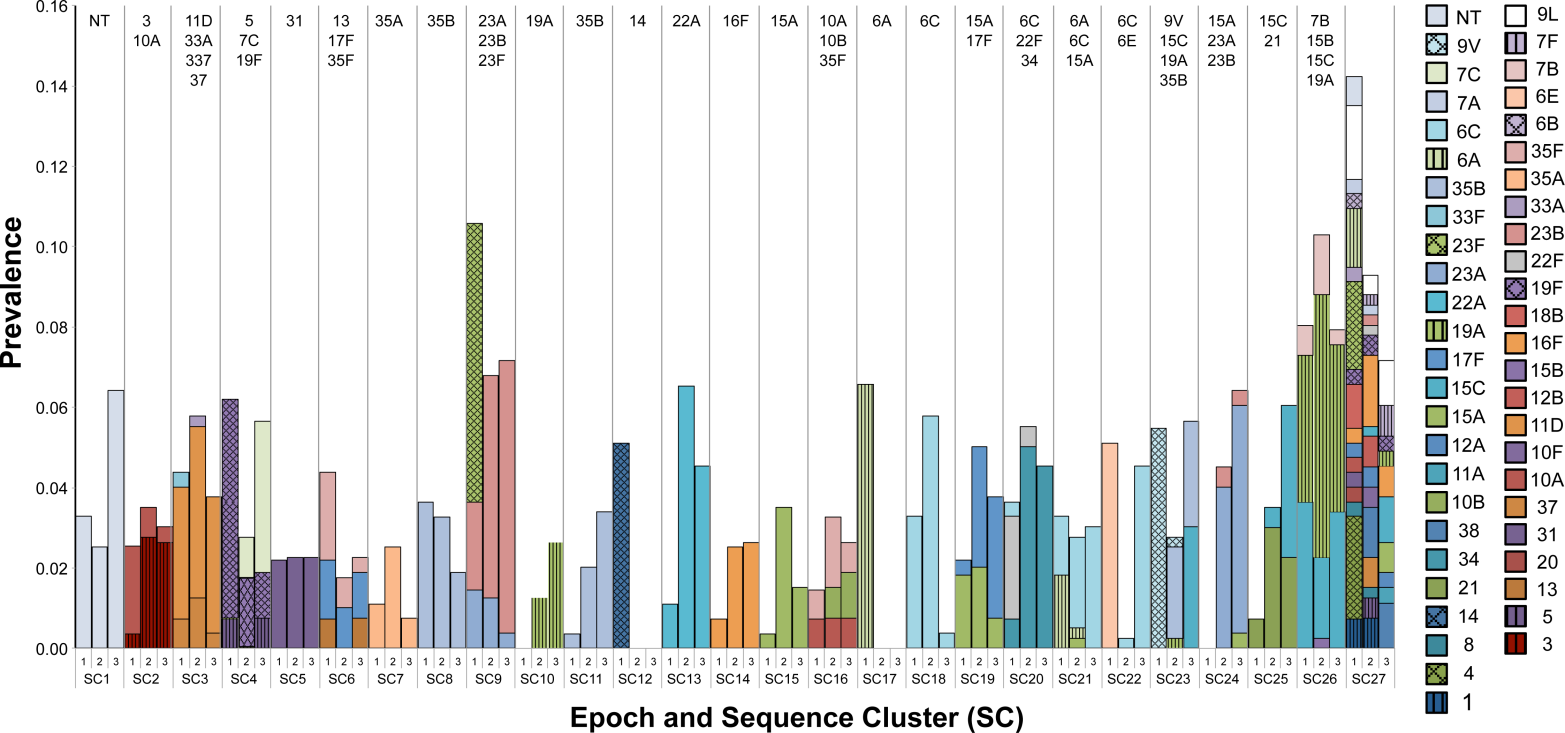
Population structure of pneumococcal carriage isolates among N/WMA. Populations are divided into sequence clusters (SC) based on genomic data and further subdivided into three epochs based on collection date. The bars represent the proportion of population comprising each SC during an epoch and are stratified by serotype composition. Solid bars represent non-vaccine serotypes; checkered hatched pattern PCV7 vaccine types; and vertical line pattern PCV13 vaccine types (those not included in PCV7). Serotypes comprising each SC are also labeled above each column. All SCs except SC4 and SC27 are monophyletic. SC4 is has three distinct sub-clades and SC27 include polyphyletic lineages present at minor frequencies in the population.

For temporal comparison, we divided study periods into three epochs and six sub-epochs (1A/B, 2A/B, 3A/B) (S2 Fig). To verify representativeness of isolates used for genome sequencing in this study, we obtained prevalence data on 3,868 carriage events from children ≤5 years of age in the parent N/WMA carriage studies from which the genomic sample was drawn. This included 1227 events from Epoch 1, 1038 from Epoch 2, and 1603 from Epoch 3. For the major epochs, the proportions of NVT, PCV7, and PCV13 serotypes in our sample were comparable with the serotype dynamics characterized by the three N/WMA parent studies (Fig 2A). The exception was the proportion of NVT and PCV7 VT in Epoch 1, which was due to differences between serological and genomic assignment of serogroup 6 isolates. In Epoch 1, serotypes 6B and 6C were both assigned to serotype 6B by the Quellung reaction used in the parent carriage study. This was subsequently resolved in the current study using a genomic approach to determine serotype, and later carriage studies were able to distinguish 6B from 6C. In pre-PCV Epoch 1, 26.3% of the sample was comprised of PCV7 VT, mostly serotypes 23F, 9V, 14, and 19F. Post-PCV7, the proportion of PCV7 VT in Epoch 2 fell to 1.8%. The prevalence of PCV13 VTs declined steadily from 17.5% in Epoch 1 to 11.3% in Epoch 3. The reduction in PCV13-specific VT after the introduction of PCV7 was likely due to the cross-reactivity of the 6B component of PCV7 with serotype 6A [61], which can be inferred from the elimination of SC17 (serotype 6A) after Epoch 1 (Fig 3).

### Population dynamics: Serotypes and lineages

Fluctuations in serotype distribution were reflected in measures of serotype diversity. Simpson’s D, which summarizes diversity as the probability that two isolates chosen at random are different, increased from Epoch 1 to 2, reflecting an increase in previously low-frequency NVT serotypes as well as the introduction of previously unobserved serotypes (Fig 2B). Fig 3 illustrates how the composition of the 27 main SCs changed during each of the three epochs. Of two lineages containing PCV7 VT only in Epoch 1, one (SC12) disappeared after vaccination, and another remained, with only PCV7 NVT isolates in Epochs 2 and 3. In SCs containing both PCV7 VT and NVT, the VT lineages were largely eliminated. After Epoch 1, the composition of the pneumococcal population in our sample and parent carriage studies shifted to a predominance of NVT and PCV13 VT, with the largest increases in serotypes 23B and 15C. While in most cases the NVT increases arose from serotypes previously observed in Epoch 1, serotypes belonging to SC10, SC22, and SC24 were not detected until Epoch 2. PCV13 VTs in our sample were not significantly impacted between Epoch 2 and 3. Further comparison of PCV13 implementation data from N/WMA communities during Epoch 3 sampling demonstrated incomplete vaccine coverage and persistence of PCV13 vaccine serotypes (S3 Fig). This finding is consistent with the previous observation that the impact of PCV13 on carriage among underimmunized children was not detected until vaccine coverage in the community reached 58% [6]. This coverage level was not attained until February 2011, at which point 52% of the Epoch 3 sample had been collected. As a result, our assessment of the impact of PCV13 on the overall pneumococcal population was limited.

### Population genetic parameters

We used Watterson’s theta (Θ_W_)–proportional to the number of polymorphic sites—and Tajima’s *D* to assess the impact of vaccine on population level genetic diversity and population size. Under neutrality and constant population size, Θ_W_ = 2*N*_*e*_*μ*, where *N*_*e*_ is the effective population size and *μ* is the mutation rate [51]. Selective removal of several clusters of related strains, such as lineages or sub-lineages associated with VT, should lead to a reduction in Θ_W_. A related measure, Tajima’s *D*, tests for evidence of population growth, with negative values suggesting population expansion (due to the presence of rare variants at high frequencies) and positive values suggesting balancing selection or population contraction [50]. Consistent with our expectations, Θ_W_ decreased from Epoch 1B to 2A, illustrating an overall decrease in pneumococcal genomic diversity, while the average number of pairwise differences (π) was unaffected (Fig 2C). Tajima’s *D* values computed for the polymorphic nucleotide sites in the core genome increased from -0.59 in Epoch 1B to 0.07 in 2A, signifying a removal of rare variants consistent with a species-wide population bottleneck (Fig 2D). By Epoch 3B both Θ_W_ and Tajima’s *D* returned to pre-PCV7 levels while π increased. No discernible changes in either measure were associated with PCV13 introduction.

### Contributions of population processes

After the population genetic bottleneck induced by PCV7’s removal of VT, genetic diversity (i.e., Θ_W_) may have been augmented by 1) clonal expansion of NVT lineages due to selection or genetic drift (to increase Θ_W_ such lineages would have to have been so rare post-bottleneck that they were not sampled), 2) introduction of new lineages, or 3) recombination. We hence examined evidence for each of these among individual SCs. Recombination rates (*r/m*) varied among SCs, ranging from 0 to 15.0, averaging 4.25 (Table 1 and S4 Fig). While coalescent analysis found SCs varied in mutation rates (S5 Fig), there was no significant difference between the median evolutionary rates of NVT and VT SCs (95% CI: -1.06e-06–8.54e-06, *F*(1,29) = 2.55, *p* = 0.12). Therefore, high evolutionary rates among NVT lineages were not solely responsible for recovering the diversity lost due to the removal of PCV7 VT.

To investigate the contribution of introduction of new lineages or expansion of previously unsampled ones, we estimated the TMRCAs (i.e., lineage age) of SCs. Overall, the median TMRCA was 1955 and ranged from 1839 (SC21: 6A/C ST473) to as recent as 2000 (SC10: 19A ST320) (S6 Fig). Two SCs that were not identified during Epoch 1 sampling emerged following vaccination: SC10 (S7 Fig), which is all type 19A and ST320, and SC24, largely comprised of serotype 23A (S8 Fig) related to PMEN clone Colombia^23F^-26. Estimated TMRCA for SC10 was 2000 [95% HPD: 1996–2004]. The lineage age, taken together with its low level of genetic diversity (Θ_w_ = 0.0006) and negative Tajima’s *D* value (-2.15), suggests that this SC was introduced after the implementation of PCV7 among southwest Native Americans and is currently experiencing population expansion. SC24 was first identified in 2006 during Epoch 2, but its most recent common ancestor was estimated at 1958 [95% HPD: 1928–1980], near the median TMRCA among all SCs. Considering its prevalence in Epoch 2 and moderate level of diversity (Θ_w_ = 0.003), it is likely that SC24 was not recently introduced and that its was present in the population before PCV7 but at a sufficiently low frequency not to be sampled until 2006, by which time its frequency may have increased. Furthermore, SC24’s low Tajima’s *D* value (-1.63) is consistent with population expansion.

### No detectable signal of vaccine impact on effective population size

We hypothesized that post-PCV7 changes in pneumococcal populations would be visible as decreases in the effective population size (*N*_*e*_) of predominantly VT lineages and increases in those of predominantly NVT lineages. The effective population size can be interpreted as the number of genomes contributing offspring to the next generation, and changes in *N*_*e*_ can be used to measure population growth or contraction. Inferring demography among SCs identified that over half (56%) fit constant population size models based on MLEs (Table 1). Furthermore, while the remainder of SCs best fit a fluctuating *N*_*e*_ model (i.e., Skygrid), assessment of *N*_*e*_ trajectories identified only three that were significantly different from a constant size based on HPDs. These three SCs (SC11, SC17, and SC26-A) were found to be decreasing throughout the study period; one was PCV13 VT (SC 17) and two were NVT (SC11 and SC 26-A). To assess bias potentially introduced by removing recombination, we tested the association between recombination rates and inferred demography, which we found to not be significant (*F*(1,30) = 0.44, *p* = 0.51) [62]. Overall, these findings show that the relatively subtle increases in sample frequencies of individual SCs containing NVT are not visible as departures from a constant *N*_*e*_.

### The impact of vaccine on the pneumococcal pangenome

**Fig 4:**
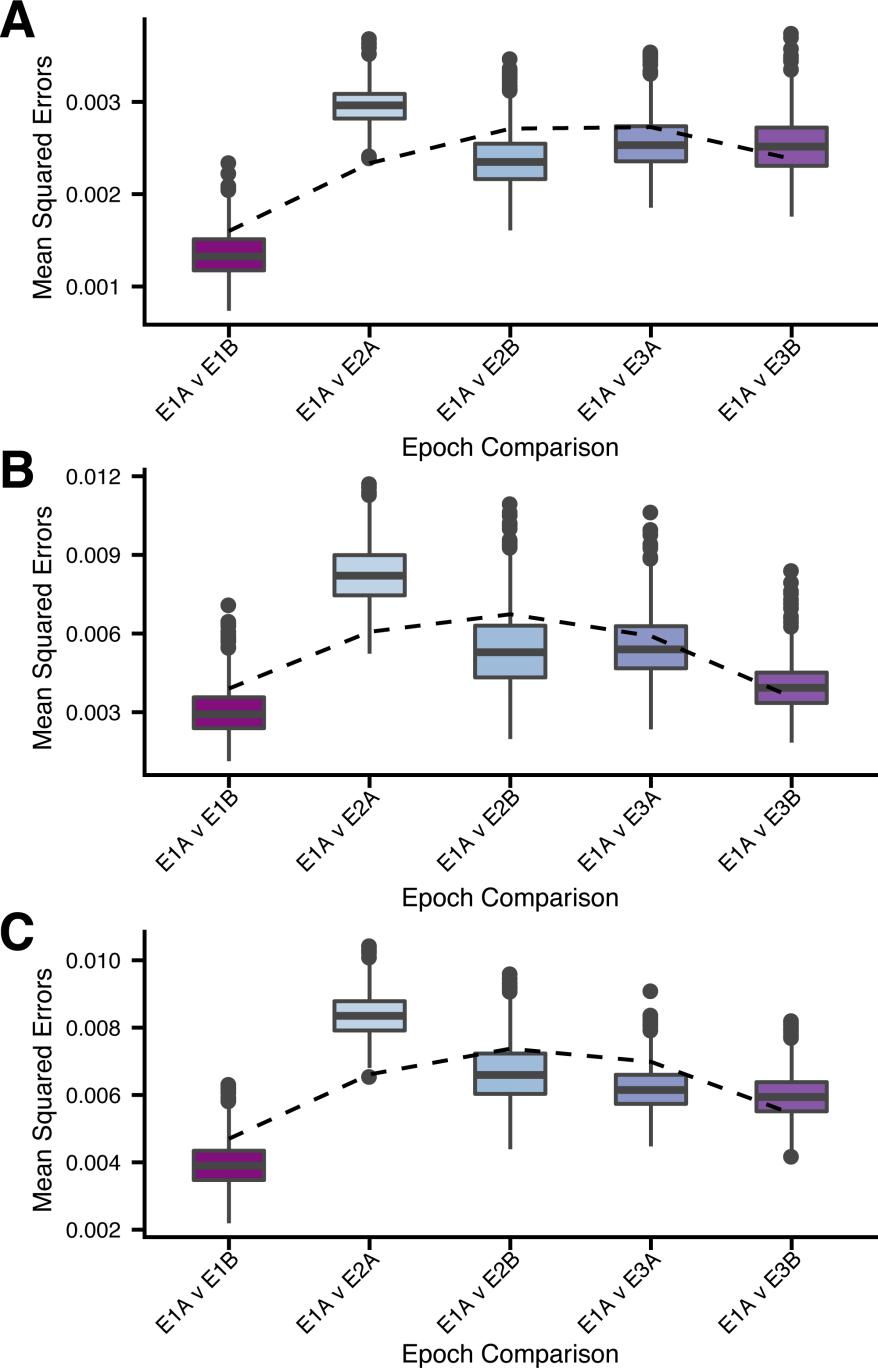
Mean squared errors (MSEs) comparing changes in genomic loci frequencies between Epoch 1A and all subsequent sub-epochs. For each comparison, 75 individuals were subsampled with replacement from each sub-epoch and 1,000 bootstrap replicates were performed. A.) MSEs for sub-epoch comparison of frequencies of 22,434 biallelic SNP sites found among 256 metabolic genes. B.) MSEs for sub-epoch comparison of frequencies of 53 variants of 19 polymorphic antigens. C.) MSEs for sub-epoch comparison of frequencies of 2370 COGs found from 5–95% among all 937 taxa.

**Fig 5:**
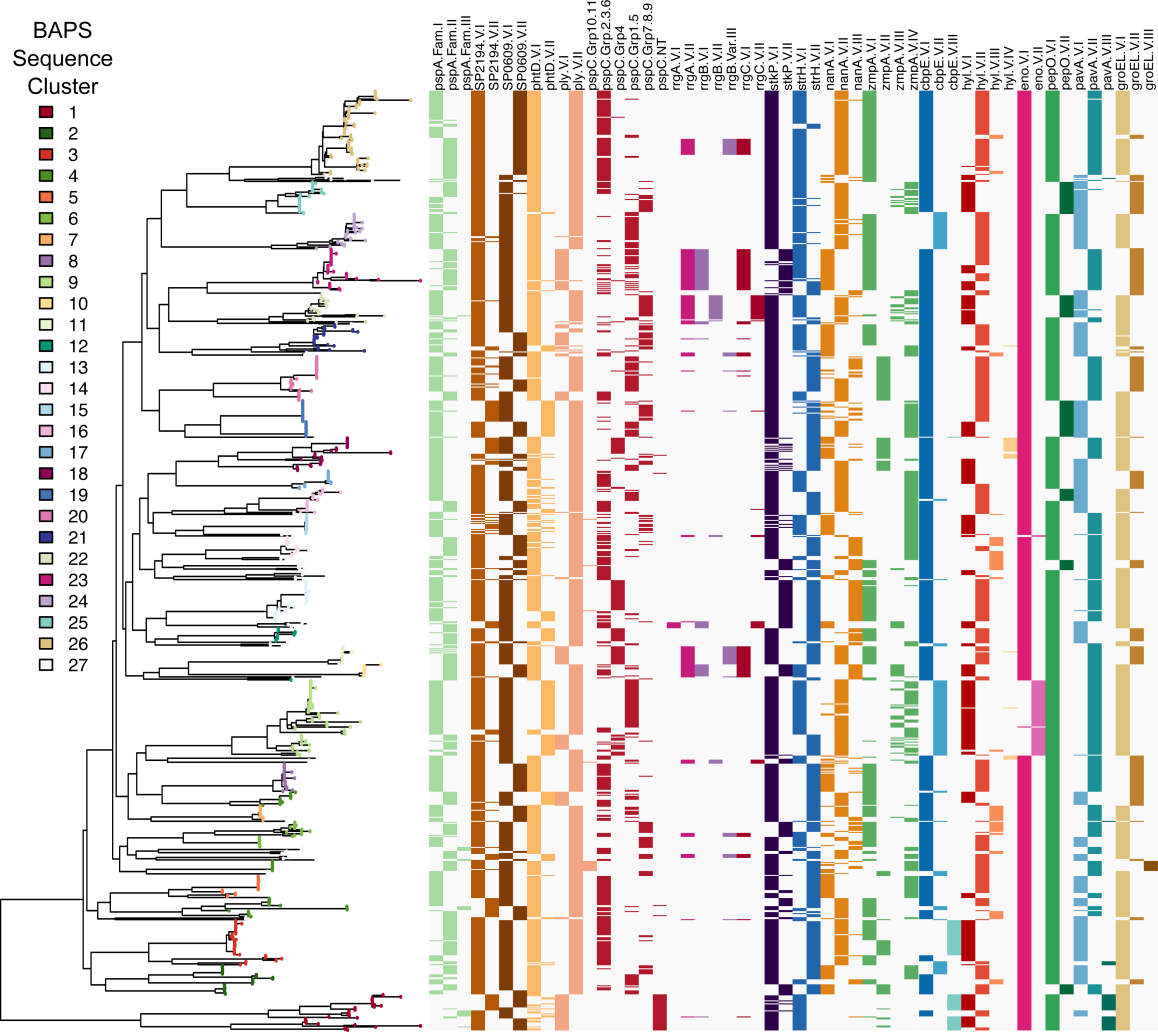
Association of hierBAPS sequence cluster (SC) with polymorphic non-capsular antigens. Maximum likelihood phylogeny of 937 pneumococcal carriage isolates corresponding to Fig 3. Color ramps in legend designate SCs. Antigens include pspA, SP2194, SP0609, phtD, ply, pspC, rrgA/B/C (type 1 pilus islet), stkP, strH, nanA, zmpA, cbpE, hyl, eno, pepO, pavA, and GroEL. Accession numbers for antigen variants have been previously published [15].

**Fig 6:**
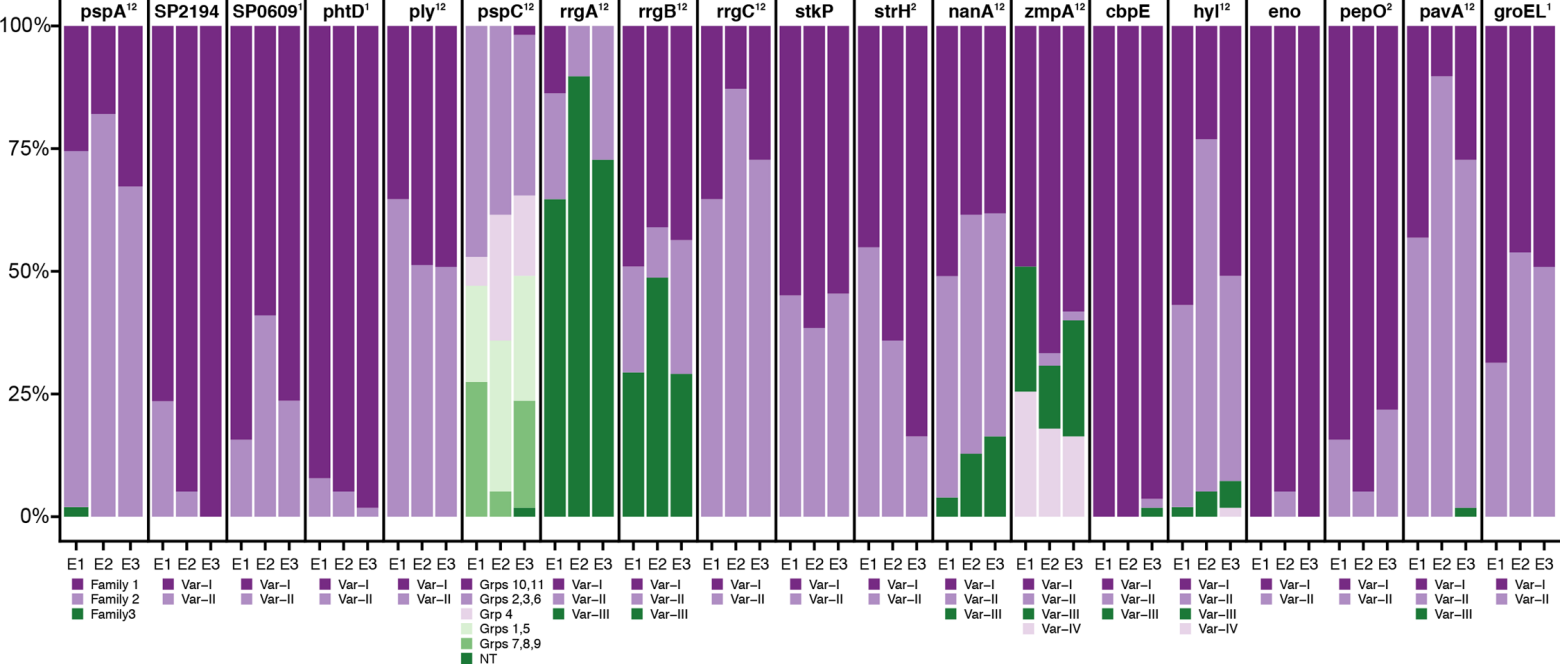
Bar plots comparing proportion of variants among 19 polymorphic antigens between Epochs E1-E3. Each plot represents the change in distribution of polymorphic antigen variants during Epoch 1 (E1), Epoch 2 (E2), and Epoch 3 (E3). Antigens are labeled above each bar plot, and variants of each antigen are colored according to the legend below each plot. Antigen labels are annotated to indicate whether the change in the distribution of frequencies was significant between E1 and E2 (“1”), E2 and E3 (“2”), or both (“12”). All antigens are found among all strains with the exception of rrgA/B/C, which are only present in a subset of taxa. Accession numbers for each variant has been previously published.

To test the hypothesis that selective removal of PCV7 VT disrupted accessory genome content, we compared accessory size and frequencies of 2370 COGs and 53 variants of 19 antigens between pre-PCV7 Epoch 1 to post-PCV7 epochs. Further, we tested the concurrent effect on metabolic loci by assessing frequencies of 22,434 biallelic SNPs found among 256 metabolic genes present in the core genome. For metabolic loci, accessory COGs, and antigen variants, within-group diversity was minimized when SC population groupings were assigned, compared to serogroup and serotype (S9 Fig). The introduction of PCV7 resulted in an overall reduction in pangenome size, illustrated by the difference in logarithmic pangenome curves for Epochs 2A and 3B (S10 Fig). A comparison of pre-PCV7 Epochs 1A and 1B provided a baseline estimate of stochastic, temporal fluctuations in frequencies in the absence of an effect of vaccine. Plotting COG frequencies in subsequent epochs demonstrated perturbation in pneumococcal accessory COGs frequencies following introduction of PCV7 (S11 Fig). This perturbation is characterized by the dispersion of frequency scatterplots comparing Epochs 1A vs. 2A [R^2^ = 0.96, MSE = 8.26x10^-3^ (95% CI: 8.32x10^-3^–8.40x10^-3^)] and 2B [R^2^ = 0.98, MSE = 6.65 x10^-3^ (95% CI: 6.60x10^-3^–6.70x10^-3^)] (Figs 4 and S11). This effect was also observed when comparing the frequencies of polymorphic antigens and metabolic loci between epochs (Figs 5, S12 and S13). For all sets of genomic loci, MSE in comparison to Epoch 1A are smaller for 1B than for any of the subsequent epochs, illustrating the disruption caused by PCV7. While this observation alone could be explained by drift leading to increasing divergence in frequencies over time, a further observation cannot: in each example, MSEs decreased from Epoch 2 to 3, indicating metabolic loci, accessory COGs, and antigen frequencies were trending back toward pre-PCV7 values (Fig 5). This trend was observed when isolates collected from individuals >5 years of age were removed from Epoch 2 and the analysis repeated. This led us to compare Epoch 3A (post-PCV7/pre-PCV13) to previous sub-epochs to determine whether the pre-PCV7 Epochs 1A/B or the immediately preceding Epoch 2B were better predictors of COG/antigen frequencies. For accessory genome COG frequencies and metabolic loci, Epoch 2B was a better predictor of 3A frequencies; however, for antigens, pre-PCV7 Epoch 1B was the best predictor of Epoch 3A frequencies (S14 Fig). Taken together, we found that antigen variant frequencies largely returned to pre-PCV7 values; however, some perturbations were not resolved (Fig 6). This was due largely to *pspC* groups 1/5 (*p* = 0.01) and *srtH* Var-I (*p* = 0.004), which remained at higher frequencies at Epoch 3, and *rrgA* Var-I (*p*<0.001), which was completely removed from the population.

## Discussion

The impact of PCV7 introduction on pneumococcal serotype distributions has been well-characterized in the N/WMA and other communities, but the pneumococcal genome-wide impact has been investigated in fewer populations [3,63]. We studied genomes from a sample spanning the introduction of PCV7 and PCV13, which, based on serotype distribution, were representative of the full set of data from which the sample was drawn. Beyond the expected impact on serotypes, we find the effect of vaccine on the pneumococcal population could be observed as changes in population level diversity, metabolic loci, size of the pneumococcal pangenome, and frequencies of accessory genes including polymorphic antigens. We further illustrate how pneumococcal genomic diversity and frequencies of accessory genome COGs rebounded after the population bottleneck induced by the selective removal of VT lineages by PCV7. These findings help explain how the frequency distribution of polymorphic antigens, for example, largely return to baseline frequencies after being disrupted by vaccine.

The post-PCV7 pneumococcal population in N/WMA saw the complete removal of two SCs and a significant reduction in prevalence of three. The population bottleneck was characterized by changes in levels and patterns of genomic diversity, decreasing Θ_W_ and increasing Tajima’s *D* (Fig 2). Subsequently, the removal of VT pneumococci was counterbalanced by the expansion of SC9 and the emergence of two previously unobserved SCs, SC10 and SC24. In Epoch 2, we identified minor variations in the distribution of SCs by age group for four SCs. As none of the SCs contained PCV7 VT, differences likely resulted from variation in acquired serotype-specific immunity among children and adults [64]. Overall, population structure of SCs was comparable, consistent with pneumococcal transmission dynamics and the wide-ranging impact of the PCV7 vaccine on carriage in children and adults [5]. Despite the changes in the prevalence of SCs over time, no consistent pattern of change in the *N*_*e*_ of these SCs was detectable through coalescent analysis of individual SCs (Table 1). This lack of signal may be due to a number of factors. It may be that where vaccine pressure was strong enough to drastically change the population size of an SC, it was eliminated (e.g., SC12), so the temporal signal was lost; where changes were more modest, e.g. in SC including both VT and NVT, the method may have been too insensitive to detect a change. While assessment of *N*_*e*_ did not clearly identify consistent changes, we did detect the post-PCV7 emergence of two SCs. By comparing TMRCA and core genome diversity, we infer that that the first, SC10, appears to have been recently introduced among N/WMA, while the second, SC24, appears to have become detectable due to the vaccine [8,65]. It is worth noting that assessing *N*_*e*_ and other population genetic parameters of pneumococcal lineages makes implicit assumptions about defining SCs as populations and a collection of SCs as a metapopulation, which, to varying degrees, may compete or interact with one another through recombination. Indeed, this definition is more complex and requires consideration of competition, gene flow, and niche overlap among lineages [60,66,67]. Here, we statistically define SCs and find that these populations are often good predictors of serogroup, metabolic profile, and gene content, thus generally demonstrate genomic coherence consistent with the concept of a bacterial population.

Pneumococcal genomic data from carriage studies in the US are limited [9]. The N/WMA sample provides an opportunity to assess post-vaccine changes in the pneumococcal populations across demographically and geographically varied regions and, at large, the generalizability of bacterial pathogen population dynamics. Comparable analysis of population structure of 616 carriage isolates from Massachusetts collected between 2001 and 2007 found less structure (15 monophyletic SCs (n = 616)) compared to the N/WMA sample (25 monophyletic SCs (n = 937)) [9], and unlike Massachusetts, where the post-PCV7 population emerged largely from the pre-existing serotype diversity, in the N/WMA sample we observed seven previously unidentified serotypes and two entire SCs post-PCV7. Considering carriage data from the larger parent studies, 13 previously unidentified serotypes, excluding 6C, were observed post-PCV7. This difference aside, SC composition and pneumococcal population dynamics were consistent between N/WMA and Massachusetts. For example, SC9 (also SC9 in the Massachusetts study [9]) experienced a near identical population shift post-PCV7 (S15 Fig). This SC, which is comprised of VT 23F and NVTs 23A and 23B, is thought to have arisen through multiple serotype-switching events. In the N/WMA sample, it was one of the most successful in terms of overall prevalence in Epoch 1. As observed in Massachusetts, PCV7 effectively removed 23F isolates from the SC; however, SC9 NVTs subsequently increased 3.5% from Epoch 1 to 3. This shows that these changes were not restricted to the Massachusetts population, but were replicated in a very different setting, and may suggest that SC9 occupies a specific niche. Consistent with this hypothesis, we find that the antigen profiles for VT 23F and the NVT 23B population that replaced it, to be largely consistent with the exception of *zmpA* (S16 Fig). Taken together, we observe similar pneumococcal population dynamics in two geographically and demographically distinct populations that share common vaccine histories, suggesting that response to population shaping processes are relatively consistent.

We find that each SC is defined by a unique profile of metabolic loci, accessory COGs, and antigen variants. These profiles are most resolved at the SC level rather than serotype or serogroup, as the same serotype can be found in multiple SCs due to switching events. Moreover, within an SC, these genomic loci show significant linkage disequilibrium despite appreciable recombination among pneumococci [68]. Consistent with this linkage, we observed a coincident impact of PCV7 on genetic diversity, accessory COG frequencies, polymorphic antigens, and metabolic loci. The population genomic perturbation that resulted from the removal of PCV7 VT was significantly mitigated by Epoch 3, with frequencies of antigen variants, in particular, returning to pre-vaccine values. A recently proposed model of NFDS provides one putative mechanism for the maintenance of antigen variants and accessory COGs at optimal frequencies [27], and variant-specific host immunity provides a biologically plausible mechanism for NFDS on antigens. Early evidence of balancing selection among pneumococci was the reemergence of strains possessing a type 1 pilus after PCV7 significantly reduced piliated serotypes [69]. In the current study, we also observe the reemergence of type 1 pilus driven by serotype 19A ST320 (SC10). And while the observation with the pilus involved a change in presence-absence frequency, we now see the same dynamic extending to frequencies of antigen variants. Yet, due to linkage it is difficult to untangle which loci are being acted upon by selection and which reflect hitchhiking. Alternatively, balancing selection could be acting upon metabolic loci which are important to niche adaption and have been implicated in post-vaccine metabolic shifts [18]. In an effort to identify which loci may be driving post-vaccine success of SCs, we considered the frequencies of metabolic loci, accessory COG, and antigen variants separately. We find PCV13-era (Epoch 3) frequencies of polymorphic antigens are better predicted by pre-PCV7 (Epoch 1) frequencies than the immediately preceding period. In addition, we observe that overall COG frequencies seemed to trend toward pre-PCV7 norms with increasing time since vaccine introduction, while frequencies of metabolic loci remained disrupted. This does not rule out variation in metabolic loci or other core genes such as GroEL as driving forces for pneumococcal population structure [70]; however, it remains difficult to assign fitness differences based on observed genetic variation. For example, two SCs may be divergent in metabolic loci but capable of exploiting the same metabolic niche.

Previous models have proposed that recombination is the mechanism underlying the post-vaccine shift in metabolic, virulence, and antigenic loci [18]. However, we argue that in our sample, recombination has likely not had enough time to shuffle antigen variants or other COGs into different genomic backgrounds. For example, if we again consider the replacement of VT 23F by NVT 23B belonging to SC9, we observe that both populations possess similar antigenic profiles (S16 Fig). Yet, the TMRCA of the 23B population, and all associated recombination events, predate the introduction of PCV7 (S15 and S16 Figs). This illustrates that at least in this case, an existing population possessing a near identical antigenic profile contributed to the rebalancing of the distribution of antigen variants in the overall pneumococcal population. Overall, the pneumococcal accessory genome is comprised of varying types of MGE (e.g., phages and antigens), and it is likely that their distribution is controlled by many different, yet interconnected, processes [17]. As such, the underlying dynamics maintaining antigenic variant and accessory COG frequencies require further investigation.

Through comprehensive analysis of serotype distribution and population dynamics of *S*. *pneumoniae* spanning the introduction of PCV7 and PCV13 among N/WMA communities, we gain a broad understanding of the impact of vaccine on population structure, serotype distribution, and pangenome composition. After the introduction of PCV7, we observe clonal replacement of VT by NVT as well as clonal expansion of vaccine-associated serotypes during a period when carriage prevalence remained unchanged. Further, we show PCV7 significantly disrupted accessory COG frequencies, including frequencies of polymorphic antigens important to host-pathogen interactions. This post-PCV7 period of ‘flux’ in serotype diversity and accessory COG distribution was normalized by Epoch 3, demonstrating rapid adaption to the post-vaccine landscape. Moving forward, continued genomic surveillance will be required to monitor the emergence of new lineages and to investigate the impact on post-PCV13 pneumococcal populations. Last, as balancing selection appears to be an integral component of pneumococcal adaption and considerable serotype-lineage-accessory genome linkage exists, the joint effect of removal of vaccine serotypes and linked antigens on host-susceptibility to extant lineages merits further study, as it has significant implications for the future of protein-based pneumococcal vaccines. For example, protein-based vaccines should consider the prevalence of polymorphic variants across host populations and either include multiple variants of the same antigen or target those in greatest frequency.

## Supporting information

S1 Fig Collection sites among Navajo and White Mountain Apache Native American communities in Southwestern United States. (EPS)

S2 Fig Samples by epoch and sub-epoch. For temporal comparison, isolates were subdivided into three epochs and six sub-epochs based on the year of collection: pre-PCV7 Epoch 1, sub-epochs A (1998) and B (1999–2001); post-PCV7 Epoch 2, sub-epochs A (2006) and B (2007–08); PCV13-Intermediate Epoch 3, sub-epochs A (2010) and B (2011–2012). An effort was made to balance the number of samples in each sub-epoch.
(EPS)

S3 Fig PCV13 vaccine coverage, proportion PCV13 VT, and isolate sampling by month and year during Epoch 3 (2010–2012). Reservation-wide PCV13 vaccine coverage (primary y-axis–black) was obtained from Indian Health Services data. Coverage is based on three doses in children 7-<12 months or 1+ dose in children from 12–59 months. The proportion of PCV13 VT carriage in children <5 years of age was calculated using data from the PCV13 parent study (n = 1,603) (primary y-axis–red). The bar-plot represents the collection month of isolates used in Epoch 3 of the present study (secondary y-axis–grey).
(EPS)

S4 Fig Recombination rate comparison among pneumococcal sequence clusters (SC). The recombination rate (*r/m*) is the ratio of SNPs predicted to have been imported through recombination events compared to those introduced through mutation. The ratio *ρ/θ* represent the relative rate of recombination events to mutations on a phylogenetic branch. The asterisks denote SCs that contain *PCV7 vaccine-types and **PCV13 vaccine types.
(EPS)

S5 Fig Evolutionary rate comparison among pneumococcal sequence clusters (SC). SC and sub-clusters are listed on the x-axis with evolutionary rates (SNPs per site per year) and 95% highest posterior density (HPD) displayed for each. Rates were inferred through population genomic modeling using BEAST v1.8.2. Demographic and molecular clock models selected through model comparison are listed in Table 1. The median evolutionary rate was 1.30E-06 SNPs/sire/year (range: 6.02E-08–7.02E-06).
(EPS)

S6 Fig Date of the most recent common ancestor (TMRCA) among pneumococcal sequence clusters (SC). TMRCA and 95% HPD were estimated using BEAST v1.8.2. Demographic and molecular clock models selected through model comparison are listed in Table 1. The median TMRCA was 1958 (range: 1839–2000).
(EPS)

S7 Fig Bayesian maximum clade credibility phylogeny of SC10. The phylogeny was inferred using BEAST v1.8.2 with relaxed molecular clock, Skygrid demographic model, and HKY nucleotide substation model. Node bars represent the 95% Highest Posterior Density (HPD) and the branch are colored based on posterior probability. A branch with a posterior probability of >0.80 is considered well supported. The density plot represents the marginal probability distribution of the root-height of the tree (i.e., TMRCA).
(EPS)

S8 Fig Bayesian maximum clade credibility phylogeny of SC24. SC24 is comprised largely of serotype 23A isolates. However, three serotype 23B1 isolates, which were basal to the 23A clade, were excluded for this analysis. Of note, among these isolates there was a serotype switch between a 23A capsule and 15A capsule. A.) The phylogeny was inferred using BEAST v1.8.2 with relaxed molecular clock, Skygrid demographic model, and HKY nucleotide substation model. Node bars represent the 95% Highest Posterior Density (HPD) and the branch are colored based on posterior probability. A branch with a posterior probability of >0.80 is considered well-supported. B.) The density plot represents the marginal probability distribution of the root-height of the tree (i.e., the most recent common ancestor (TMRCA)). C.) Bayesian Skygrid plot of effective population size (log *Ne*) over time. The grey area represents the 95% HPD of the *Ne* estimate. While effective population size appears to be increasing over time, the increase is not significant based on the HPD range.
(EPS)

S9 Fig Comparison of population stratifications and within-group genetic distance. Within-grouping genetic distance, measured as the patristic distance between pairs of isolates in the maximum likelihood (ML) phylogeny, between three population stratifications: serogroup, serotype, and sequence cluster (SC). ML phylogenies were inferred from 1) presence absence-alignment of 2371 accessory genome clusters of orthologous genes (COGs), 2) 22,424 biallelic polymorphic sites found in 272 metabolic genes present in the core genome, and 3) presence-absence alignment of non-capsular antigen variants. Error bars represent the weighted mean plus or minus the weighted standard error of the estimate. In all instances, within-group distances were significantly less than between-group mean distances.
(EPS)

S10 Fig Pangenome comparison of epochs and sub-epochs. Pangenome comparison of strains from respective epochs are overlaid, demonstrating variation in accessory clusters of orthologous genes (COG) content after introduction of PCV7. Dotted lines represent the increase in accessory COGs as the number of genomes is increased. Epochs are individually colored. The trajectory of the “total genes” reflects the overall diversity of COGs during each epoch. E1A and E1B represent the pre-PCV7 COG diversity.
(EPS)

S11 Fig Scatterplots comparing accessory genome clusters of orthologous genes (COG) frequencies among epochs. Each plot represents a comparison of COG frequencies during Epoch 1 compared to subsequent epochs. As a control, pre-PCV7 Epochs 1A and 1B are compared to each other. The sum of residuals was obtained from regressing the COG frequencies of one epoch on another using a linear regression model. Pearson’s correlation coefficients are also presented for each comparison.
(EPS)

S12 Fig Scatterplots comparing metabolic loci frequencies among epochs. Each plot represents a comparison of frequencies of 22,434 biallelic SNPs found among 256 metabolic genes present in the core genome during Epoch 1 compared to subsequent epochs. As a control, pre-PCV7 Epochs 1A and 1B are compared to each other. The sum of residuals was obtained from regressing the frequencies of one epoch on another using a linear regression model. Pearson’s correlation coefficients are also presented for each comparison.
(EPS)

S13 Fig Scatterplots comparing polymorphic protein antigen variant frequencies (n = 19) among epochs. Each plot represents a comparison of protein antigen variants frequencies during Epoch 1 compared to subsequent epochs. As a control, pre-PCV7 Epochs 1A and 1B are compared to each other. Variants of each polymorphic protein antigen are colored similarly. The sum of residuals was obtained from regressing the variant frequencies of one epoch on another using a linear regression model. Pearson’s correlation coefficients are also presented for each comparison.
(EPS)

S14 Fig Predictability of Epoch 3 genomic loci frequencies by previous sub-epochs. Overlaid histograms of mean squared errors (MSEs) from 1,000 bootstrap replicates comparing three sets of genomic loci frequencies between Epoch 3A and preceding sub-epochs 1B, 2A, and 2B. Histograms are colored according to the epoch comparison. Dashed lines represent median MSE values for each comparison. MSE values closer to zero indicated better prediction of Epoch 3A frequencies. A.) MSEs for sub-epoch comparison of frequencies of 22,434 biallelic SNP sites found among 256 metabolic genes. B.) MSEs for sub-epoch comparison of frequencies of 53 variants of 19 polymorphic, non-capsular antigens. For protein antigens, Epoch 3A vs. 1B frequencies had lower MSEs [5.78x10^-3^ (SE 3.92x10^-5^)] than Epoch 3A vs. 2B [6.06x10^-3^ (SE 5.45x10^-^)], indicating E1B pre-PCV7 frequencies were better predictors of post-PCV7 frequencies that than the immediately preceding period (E2B). C.) MSEs for sub-epoch comparison of frequencies of 2370 COGs found from 5–95% among all taxa (n = 937).
(EPS)

S15 Fig Bayesian maximum clade credibility phylogeny of SC9. SC9 is comprised of serotypes 23B, 23F, and 23A. A.) The phylogeny was inferred using BEAST v1.8.2 with relaxed molecular clock, Skygrid demographic model, and HKY nucleotide substation model. Node bars represent the 95% Highest Posterior Density (HPD) and the branch are colored based on posterior probability. A branch with a posterior probability of >0.80 is considered well supported. B.) The density plot represents the marginal probability distribution of the root-height of the tree (i.e., the most recent common ancestor (TMRCA)). C.) Bayesian Skygrid plot of effective population size (log *Ne*) over time, and the grey area represents the 95% HPD of the *Ne* estimate. The effective population size remained constant over the study period.
(EPS)

S16 Fig Comparison of polymorphic protein antigens and recombination events among VT and NVT serotypes comprising heirBAPS sequence cluster (SC) 9. Maximum likelihood phylogeny of 75 pneumococcal serogroup 23 carriage isolates. Heatmap illustrates the serotype and protein antigens profile including pspA, SP2194, phtD, pspC, stkP, strH, and nanA. Accession numbers for protein variants have been previously published [47]. Recombination events are presented in the right half of the figure, visualized linearly in reference to the D39 genome. VT serotype 23F was replaced by NVT serotype 23B. While 23A isolates have a varying protein antigen profile, 23F and 23B are largely comparable. Of note, multiple recombination events within this SC resulted in variations in protein antigen profile composition; however, recombination events on the major 23B clade predate the introduction of PCV7.
(EPS)

S1 Table Table of accession numbers and associated metadata. (XLSX)
